# Risk assessment of aflatoxin in Iowa corn post‐harvest using an event tree analysis: A case study

**DOI:** 10.1111/risa.15074

**Published:** 2024-07-21

**Authors:** Emily Branstad‐Spates, Gretchen A. Mosher, Erin Bowers

**Affiliations:** ^1^ Department of Agricultural and Biosystems Engineering Iowa State University Ames Iowa USA

**Keywords:** aflatoxin mitigation, event tree analysis, feed safety

## Abstract

Mycotoxins are secondary metabolites produced by fungi found in corn and are anticipated to increase globally due to enhanced weather extremes and climate change. Aflatoxin (AFL) is of concern due to its harmful effects on human and animal health. AFL can move through complex grain supply chains in the United States, including multiple stakeholders from farms, grain elevators, grain and ethanol processors, and feed mills, before reaching end users, putting numerous entities at risk. Since corn is an essential food and feed product, risk management of AFL must be considered. This case study aimed to (1) calculate the probabilities of pivotal events with AFL in corn at Food Safety Modernization Act‐regulated entities using an event tree analysis (ETA) and (2) propose recommendations based on factors identified through the ETA for AFL risk management. The ETA was based on historical AFL prevalence data in Iowa above a 20‐part per billion (ppb) threshold (2.30%). Results showed four single‐point failures in feed safety systems, where countermeasures did not function as designed. Failure is defined as the type 2 error of corn being infected with AFL <20 ppb, when it is >20 ppb, and the overall system fails to detect this with contaminated corn reaching end users. The success rate is defined as detecting the corn samples correctly >20 ppb. The average success rate was 50.14%, and the failure rate was 49.86%. It was concluded that risk‐informed decisions are a critical component of effective AFL monitoring in corn, with timely intervention strategies needed to minimize the overall effects on end users.

## 1 INTRODUCTION

Food and feed safety costs the United States ∼$110 billion annually in lost productivity and medical expenses (WHO, 2020). Among food and feed safety concerns, mycotoxins are rated the highest chronic health risk for feed products (Battilani & Leggieri, 2015; Kuiper‐Goodman, 2004). Mycotoxin detection and mitigation are estimated at $418–$1.66 billion annually in the United States, equaling a large percentage of food and feed safety issues' overall economic impact (Mitchell et al., 2016; Vardon et al., 2003; Wu, 2006). Along with a significant economic impact, aflatoxin (AFL) prevalence rates are anticipated to increase in future years (Mitchell et al., 2016; Yu et al., 2022). With climate change increasing temperatures and extreme weather events for corn production, the potential for greater fungi presence with increased AFL contamination can threaten US food and feed security and safety (Ortiz‐Bobea et al., 2021; Wu et al., 2011). Yu et al. (2022) predicted that 89.50% of corn‐growing counties, including the Corn Belt and high‐producing corn states like Iowa, will experience an increase in AFL contamination levels in 2031–2040 compared to 2011–2020.

Action levels have been imposed by the Food and Drug Administration (FDA) to minimize AFL in the corn supply chain, with industry guidelines for other mycotoxins (FDA, 2022). The FDA standard is 20 parts per billion (ppb) for human food and animal feeds (corn and other grains) for immature animals or unknown destinations of AFL (FDA, 2019). Despite implementing FDA regulations in the feed and food industry, the creation of the Food Safety Modernization Act (FSMA) in 2011, and current good agricultural and manufacturing practices (cGMPs), US mycotoxin management programs do not ensure the avoidance of AFL in corn, as corn originates in the field during the growing season, where farmers are exempt from FDA jurisdiction (Jard et al., 2011). The first entity to assume control of AFL‐affected grain is liable for downstream food and feed safety issues; therefore, gain handlers, processors, and end users need practical guidance on proactively monitoring and managing AFL with inbound corn (Fumagalli et al., 2021; C. Hurburgh & Bowers, 2015).

This study proposes a novel methodology using an event tree analysis (ETA) to assess the exposure of AFL in corn and calculate the probabilities of decision paths in FSMA‐regulated entities. FSMA‐regulated entities are defined as businesses that manufacture, process, pack, or hold food for consumption for animals in the United States. These include feed mills, animal feed and pet food manufacturers, processors, and holding facilities (FDA, 2024). Farmers that grow crops for animal food and vertically integrated farming operations are exempt from the FSMA, as well as retail establishments that sell animal feed directly to consumers and home‐based producers (FDA, 2024). The specific objectives of this study were to (1) identify and calculate the probabilities of pivotal events with AFL in corn at FSMA‐regulated entities with an ETA and (2) propose recommendations based on factors identified through the ETA and historical Iowa mycotoxin contamination data for AFL risk management. In this case study, the risk level of AFL‐contaminated grain was quantified using historical aflatoxin contamination data in Iowa over 5 years above the 20‐ppb regulatory limit imposed by the FDA (23/1006 data points overall; 2.30%; Branstad‐Spate, Castano‐Duque, et al., 2023). An improved understanding of AFL risk in feed safety systems facilitates can enhance preparation by supply chain participants to prepare them for future outbreak events.

## 2 MATERIALS AND METHODS

### 2.1 Event tree analysis

ETA is an analytical technique used to evaluate processes and events leading to a potential outcome or failure within a given system (Andrews & Dunnett, 2000). It uses binary logic, where an initiating event (IE) or set of conditions occurs, and subsequent events follow, determining outcomes (Ostrom & Wilhelmsen, 2019). The event may trigger countermeasures to prevent a mishap from occurring or propagating within a given system, where it can succeed or fail (Andrews & Dunnett, 2000). ETA is considered a bottom‐up, deductive analytical approach useful for decision‐making and risk analysis in management systems (Andrews & Dunnett, 2000; Wang & Ruxton, 1997).

### 2.2 Data collection

Limited literature has been published with risk assessment tools, including an ETA with AFL, to determine the probabilities of events at FSMA‐regulated facilities (Gupta et al., 2022). Therefore, the quantitative analysis in this study uses probabilities to estimate these pivotal events to determine the outcome event probability or frequency in the event tree (Ferdous et al., 2009; Kenarangui, 1991; Lee et al., 2018). Data used to estimate the likelihood of pivotal events were based on quantitative and qualitative data from previously published literature, expert knowledge, and practical observations from stakeholders within the integrated feed industry regarding AFL in corn (Ferdous et al., 2009). Contamination of AFL on an annual basis in corn was calculated using historical AFL contamination data from the Iowa Department of Agriculture and Land Stewardship, including 1006 data points. The AFL data used a risk threshold (severity × probability), where it categorized two contamination levels for AFL (high > 20 ppb and low < 20 ppb) (Branstad‐Spates, Castano‐Duque, et al., 2023). Based on risk contamination levels, the prevalence rates for high AFL events were calculated at 2.30% (23 high data points; Branstad‐Spates, Castano‐Duque, et al., 2023). The ETA was constructed with alternating pivotal events if AFL were contaminated at levels above or below FDA's regulatory threshold (>20 ppb) at FSMA‐regulated facilities (FDA, 2022).

Farmers are exempt from FSMA; therefore, it was an assumption in the model that farmers thought the corn to be below the regulatory limit of <20 ppb for AFL when it arrived at the point of first receipt (grain elevator, feed mill, or feed/food processor). The beginning of the ETA initiated at an FSMA‐regulated facility. A critical component of AFL contamination outcomes and potentially entering the feed and food supply chain is preventative controls qualified individuals (PCQI) and employee decision‐making. Data used for the input variable were drawn from earlier research based on the decision to sample and test corn for AFL at the point of first receipt at a grain elevator (Bowers & Mosher, 2022; Krska et al., 2005). Alongside employee decision‐making, sampling for AFL in corn is the largest source of error and variability due to the collection procedure (Whitaker, 2006; Whitaker et al., 1974). A small percentage of kernels is contaminated, and the contamination of a single kernel can be high, making it difficult to obtain a representative sample (Janik et al., 2021; Whitaker, 2006). Sampling error was considered an additional pivotal event in the ETA outside human decision‐making (Barr et al., 2021; C. Hurburgh, 2012; Whitaker et al., 1974). The testing error was constructed in the ETA to account for analytical error (false positives and negatives) of lateral flow immunoassays (Johansson et al., 2000; Lattanzio et al., 2019; Zhao et al., 2021). An assumption was made that when samples are collected, the sampling and testing completed by the employee are checked and validated by a supervisor or a PCQI (Mosher et al., 2013). Based on these results for AFL, employees and/or PCQI are faced with a secondary decision of accepting or rejecting a load of corn into the facility, resulting in another operator decision (Mosher et al., 2013). An assumption was added that AFL >20 ppb cannot be legally blended to a minimize concentration levels.

From this point forward, the remaining ETA was based on mitigating barriers and countermeasures within FSMA‐regulated entities (grain elevators, feed processors, and feed mills) to determine the outcome if AFL was present above regulatory limits (>20 ppb). It is important to note that FSMA‐regulated entities were grouped in the ETA; however, these are often stand‐alone facilities with differing risk mitigation procedures. The mitigating barriers were chosen from previous literature and expert knowledge. The first mitigating barrier is whether the facility can segregate AFL‐contaminated grain for storage and enhanced aeration (Channaiah & Maier, 2014). Most elevators can segregate two to three loads of contaminated grain and are used as a standard for Iowa facilities as an assumption in the ETA (C. Hurburgh, n.d.; C. R. Hurburgh, 1994). The second mitigating barrier measures whether the facility can physically reduce the concentration levels with cleaning tactics by removing damaged kernels, fines, and screenings (Channaiah & Maier, 2014; Coradi et al., 2016; Pascale et al., 2020; Sipos et al., 2021; Sumner & Lee, 2009; Yoder et al., 2017). Lastly, there is much published research on the detoxification tactics used for AFL in corn. The last pivotal event was the detoxification of AFL based on physical, chemical, or biological additives/processing techniques as an average across methods (Branstad, 2019; Sipos et al., 2021). If the detoxification tactics were separated based on the method, the ETA would have been too large; therefore, it was combined for this analysis.

The personal and systemic factors formed the basis of the event sequences, and the probability was calculated based on the success and failure of each pivotal event (Mosher & Keren, 2011). Based on the outcomes of events, strategies to manage AFL were created as guidelines for the industry and to support quick decision‐making as part of an AFL monitoring program that can be implemented in FSMA‐regulated facilities. The construction of the ETA is described in the following paragraphs.

### 2.3 Construction of event tree analysis

In this case study, the IE assumes that AFL prevalence rates of 2.30% for corn at the point of first receipt in an FSMA‐regulated entity in Iowa is above the 20‐ppb action limit set by the FDA (Branstad‐Spates, Castano‐Duque, et al., 2023). The second step begins by asking how the existing feed safety systems work through pivotal events. From there, alternate logic sequences are used, where the success or “yes” path works as designed. The second path is a failure or “no,” where the countermeasures do not function as designed to stop the undesirable outcome. In this research, AFL‐contaminated grain reaching undesirable end users above regulatory limits is a failure. The following steps for ETA construction involve probability calculations (Ferdous et al., 2009). The probabilities of the logic paths were calculated using standard ETA notation found in Table 1. The likelihood of each contamination path was calculated, with the probabilities of failure and success paths determined by adding all probabilities together for each type of path based on the Boolean logic (Andrews & Dunnett, 2000; Ericson, 2015; Mosher & Keren, 2011). The hypothetical probabilities assigned to the system's countermeasures are shown in Table 2.

**TABLE 1:** Logic path probability formulas.

Event	Domain	Equation
Initiating event: AFL in Iowa corn > 20 ppb (*P*_ie_)	NA	*P*_ie _= 0.023
Employee samples for AFL following the protocol (*P*_protocol_)	Yes (success)	*P*_ie_ *P*_protocol_
Employee does not sample for AFL via the protocol (1 − *P*_protocol_)	No (failure)	*P*_ie_(1 − *P*_protocol_)
AFL correctly sampled (*P*_sampling_)	Yes (success)	*P*_ie_ *P*_protocol_ *P*_sampling_
AFL not correctly sampled (1 − *P*_sampling_)	No (failure)	*P*_ie_ *P*_protocol_ (1 − *P*_sampling_)
AFL test is accurate (*P*_testing_)	Yes (success)	*P*_ie_ *P*_protocol_ *P*_sampling_ *P*_testing_
AFL test not accurate: false positives or negatives (1 − *P*_testing_)	No (failure)	*P*_ie_ *P*_protocol_ *P*_sampling_ (1 − *P*_testing_)
PCQI validates test results (*P*_validation_)	Yes (success)	*P*_ie_ *P*_protocol_ *P*_sampling_ *P*_testing_ *P*_validation_
PCQI does not validate test results (1 − *P*_validation_)	No (failure)	*P*_ie_ *P*_protocol_ *P*_sampling_ *P*_testing_ (1 − *P*_validation_)
Employee rejects load based on test results (*P*_rejects_)	Yes (success)	*P*_ie_ *P*_protocol_ *P*_sampling_ *P*_testing_ *P*_validation_ *P*_rejects_
Employee incorrectly accepts load into the facility (1 − *P*_rejects_)	No (failure)	*P*_ie_ *P*_protocol_ *P*_sampling_ *P*_testing_ *P*_validation_ (1 − *P*_rejects_)
AFL corn is segregated into another bin (*P*_segregates_)	Yes (success)	*P*_ie_ *P*_protocol_ *P*_sampling_ *P*_testing_ *P*_validation_ (1 − *P*_rejects_) *P*_segregates_
AFL corn is not segregated into another bin (1 − *P*_segregates_)	No (failure)	*P*_ie_ *P*_protocol_ *P*_sampling_ *P*_testing_ *P*_validation_ (1 − *P*_rejects_) (1 − *P*_segregates_)
AFL corn is cleaned and/or dried (*P*_cleans_)	Yes (success)	*P*_ie_ *P*_protocol_ *P*_sampling_ *P*_testing_ *P*_validation_ (1 − *P*_rejects_) *P*_segregates_ *P*_cleans_
AFL corn is not cleaned and/or dried (1 − *P*_cleans_)	No (failure)	*P*_ie_ *P*_protocol_ *P*_sampling_ *P*_testing_ *P*_validation_ (1 − *P*_rejects_) *P*_segregates_ (1 − *P*_cleans_)
AFL is detoxified by physical, chemical, or biological methods after cleaning (*P*_detoxify_)	Yes (success)	*P*_ie_ *P*_protocol_ *P*_sampling_ *P*_testing_ *P*_validation_ (1 − *P*_rejects_) *P*_segregates_ *P*_cleans_ *P*_detoxify_
AFL is not detoxified by physical, chemical, or biological methods after cleaning (1 − *P*_detoxify_)	No (failure)	*P*_ie_ *P*_protocol_ *P*_sampling_ *P*_testing_ *P*_validation_ (1 − *P*_rejects_) *P*_segregates_ *P*_cleans_ (1 − *P*_detoxify_)
System failure probability	Overall system failure	[*P*_ie_(1 − *P*_protocol_)] + [*P*_ie_ *P*_protocol_ (1 − *P*_sampling_)] + [*P*_ie_ *P*_protocol_ *P*_sampling_ (1 − *P*_testing_)] + [*P*_ie_ *P*_protocol_ *P*_sampling_ *P*_testing_ (1 − *P*_validation_)] + [*P*_ie_ *P*_protocol_ *P*_sampling_ *P*_testing_ *P*_validation_(1 − *P*_rejects_) (1 − *P*_segregates_)] + [*P*_ie_ *P*_protocol_ *P*_sampling_ *P*_testing_ *P*_validation_ (1 − *P*_rejects_) *P*_segregates_ *P*_cleans_ (1 − *P*_detoxify_)] + [*P*_ie_ *P*_protocol_ *P*_sampling_ *P*_testing_ *P*_validation_ (1 − *P*_rejects_) *P*_segregates_ (1 − *P*_cleans_) (1 − *P*_detoxify_)]
System success probability	Overall system success	[(*P*_ie_ *P*_protocol_ *P*_sampling_ *P*_testing_ *P*_validation_ *P*_rejects_)] + [(*P*_ie_ *P*_protocol_ *P*_sampling_ *P*_testing_ *P*_validation_ (1 − *P*_rejects_) *P*_segregates_ *P*_cleans_ *P*_detoxify_ *P*_ie_ *P*_protocol_ *P*_sampling_ *P*_testing_ *P*_validation_ (1 − *P*_rejects_) *P*_segregates_ *P*_cleans_ *P*_detoxify_)] + [(*P*_ie_ *P*_protocol_ *P*_sampling_ *P*_testing_ *P*_validation_ (1 − *P*_rejects_) *P*_segregates_ (1 − *P*_cleans_) *P*_detoxify_)]

Abbreviations: AFL, aflatoxin; NA, not available; PCQI, preventative controls qualified individual.

**TABLE 2:** Possible decision and system responses for aflatoxin (AFL) contamination in Food Safety Modernization Act (FSMA)‐regulated entities.

Decision choice or countermeasure	Probability^a^	Range	Reference
AFL > 20 ppb (IE)	IE = 0.023	N/A	♦ Branstad‐Spates, Castano‐Duque, et al., 2023
Employee decides to sample for AFL	Yes = 0.86	N/A	♦ Bowers & Mosher, 2022
	No = 0.14	(survey data)	
Representative sample obtained	Yes = 0.67	Yes = 60%–75%	♦ C. Hurburgh, 2012
	No = 0.33	No = 25%–45%	♦ Whitaker et al., 1974
			♦ Whitaker, 2006
Testing detects AFL	Yes = 0.98	Yes = 95%–99.9%	♦ Johansson et al., 2000
	No = 0.02	No = 0.1%–2%	♦ Lattanzio et al., 2019
			♦ Lupo et al., 2010
			♦ Zhao et al., 2021
PCQI validates sampling and testing for AFL	Yes = 0.90	N/A	Assumption in the model by expert opinion
	No = 0.10	(expert opinion)	
Employee accepts or rejects the load	Yes = 0.96	N/A	♦ Mosher et al., 2013
	No = 0.04	(survey data)	
Segregation of AFL‐contaminated corn	Yes = 0.98	N/A	♦ C. R. Hurburgh, 1994
	No = 0.02	(expert opinion)	♦ Assumption in the model by expert opinion
Cleaning or drying of corn	Yes = 0.56	Yes = 26%, 50%, 65%–84%	♦ Pascale et al., 2020
	No = 0.44	No = 16%–36%	♦ Sipos et al., 2021
			♦ Sumner & Lee, 2009
			♦ Yoder et al., 2017
Chemical, physical, or biological detoxification techniques	Yes = 0.68	Yes = 51%–85%	♦ Branstad, 2019
	No = 0.32	No = 15%–39%	♦ Sipos et al., 2021
Overall system success	*p* = 0.011532		50.14%
Overall system failure	*p* = 0.011459		49.86%

Abbreviations: IE, initiating event; N/A, not available; PCQI, preventative controls qualified individual; ppb, parts per billion.

^a^ Probabilities are based on previous literature, expert knowledge, and practical observation. These values are averages from the given ranges.

## 3 RESULTS

### 3.1 Probability estimation

**FIGURE 1:**
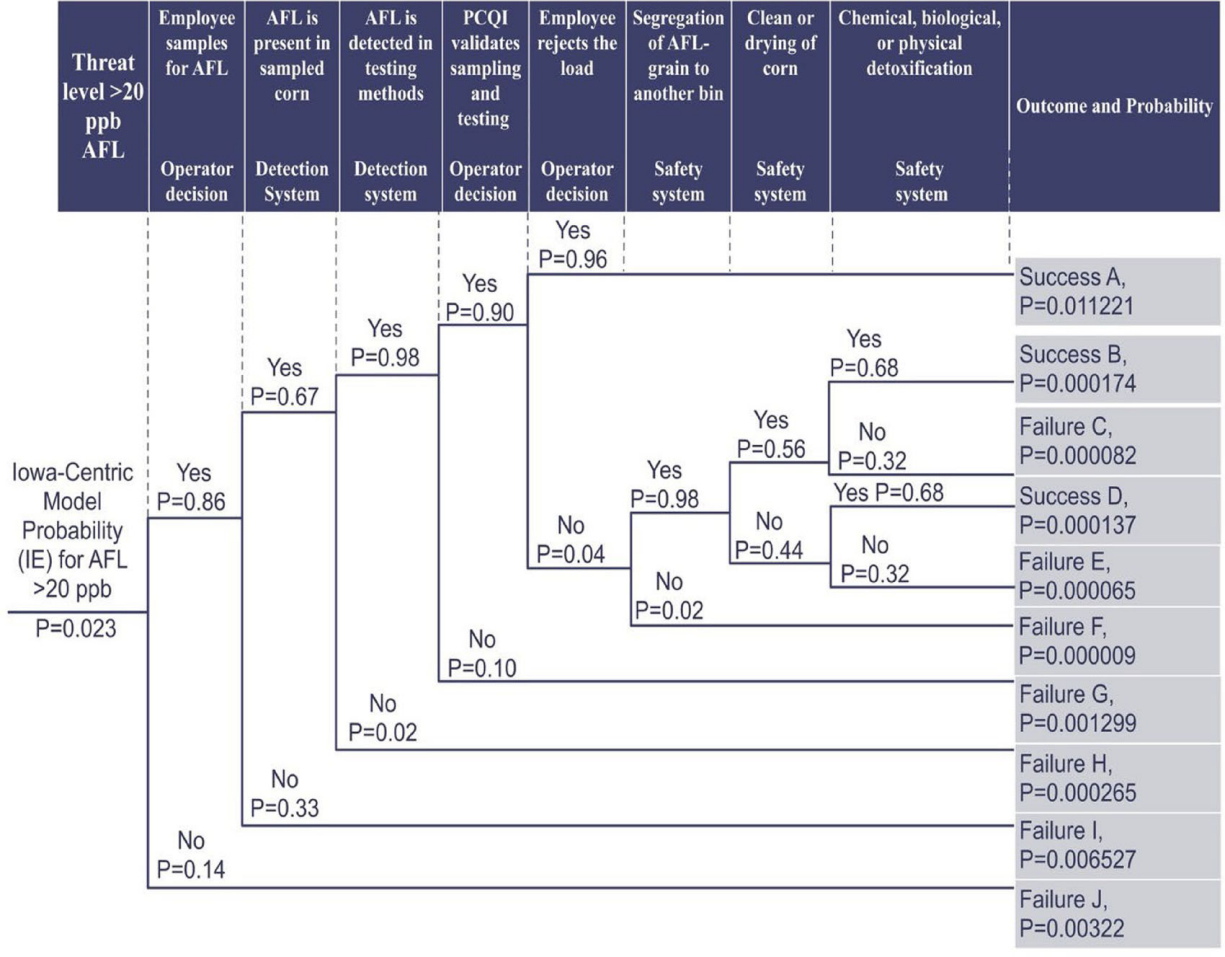
Event tree analysis (ETA) for aflatoxin (AFL) in a hypothetical scenario for a Food Safety Modernization Act (FSMA)‐regulated entity in the United States. IE, initiating event; PCQI, preventative controls qualified individual; ppb, parts per billion.

The probability of the intiating event for an AFL‐contamination event in Iowa (>20 ppb) was assigned as 2.30%, based on a 5‐year average noted by previous researchers (Branstad‐Spates, Castano‐Duque, et al., 2023). Given the probabilities of pivotal events, the success decision paths were calculated accordingly. The probability of the failure paths was calculated by subtracting the opposite success paths from the IE probability. The probabilities were calculated at each decision choice or countermeasure and multiplied to determine each path's probability. To estimate success and failure probabilities within the overall system, the probabilities of all the paths were summed together. The ETA with the calculated events, failures, and success paths are shown in Figure 1.

From the estimates in Table 2, the probability of system success was calculated to be 50.14%, and the probability of system failure was 49.86%. Based on these probability estimates, the ETA suggests that feed systems with FSMA regulation have approximately a 50/50 chance of mitigating AFL from the supply chain. There were approximately four single‐point failures (SPF) in the ETA, defined as when one portion of the system fails, and there is a complete failure in the overall system (Andrews & Dunnett, 2000). The largest SPF in the ETA was from sampling, followed by employees deciding to test for AFL, validation of sampling and testing, and testing methodology. The control points determined for AFL control from the ETA were sampling and human decision‐making with inbound corn.

### 3.2 Strategies to manage AFL post‐harvest

Based on the analysis conducted in this research, a comprehensive checklist to manage AFL post‐harvest relevant to areas that need to be controlled due to failures in feed systems is listed in Table 3. These risk management strategies represent best practices to mitigate or control AFL in prior literature, and several well‐known strategies have been published (Fumagalli et al., 2021). The four supply chain stages strategies to manage AFL, excluding pre‐harvest and harvest, are at the grain elevator, grain processor, feed mill, and end user.

**TABLE 3:** Strategies to manage aflatoxin (AFL) post‐harvest in supply chains. Adapted from Fumagalli et al. (2021).

Feed cycle phase	Strategies to manage AFL	Reference
Point of first receipt for corn at the grain elevator	♦ Quality control of corn at intake: check for moisture below 15%, insect damage, and damaged kernels. ♦ AFL monitoring strategy is based on the given location's risk assessment outputs (sampling frequency and testing). ♦ Training of employees and PCQI for sampling, testing preparation, and analysis. ♦ Grain aeration, ventilation, and movement of contaminated corn based on test results and moisture content.	♦ Binder, 2007 ♦ Channiah & Maier, 2014 ♦ Coker et al., 1984 ♦ Fumagalli et al., 2021 ♦ Lopez et al., 1999
Storage of corn at the grain elevator	♦ Cleaning and sanitation of facilities based on cGMPs (prevent rodents, birds, insects, dust, and debris). ♦ Application of mold inhibitors, if necessary, based on test results and end users. ♦ Control abiotic factors (water, air temperature) and biotic factors (corn, bacteria, yeast, and fungi) with monitoring and aeration systems, lowering the internal temperature in the bin, etc. ♦ Correct handling of waste.	♦ Chulze, 2010 ♦ Fumagalli et al., 2021 ♦ Magan & Aldred, 2007 ♦ Mannaa & Kim, 2017
Transportation	♦ Regularly clean vehicles to prevent cross‐contamination of AFL‐contaminated grain. ♦ Implement a validated cleaning protocol in between the loads. ♦ Maintain records of all movements of AFL‐contaminated grain.	♦ Hernandez‐Nopsa et al., 2015 ♦ Fumagalli et al., 2021 ♦ Nada et al., 2022 ♦ Neme & Mohammad, 2017
Intake of corn at the grain processor or feed mill	♦ Maintain corn processing and testing before acquisition. ♦ Sample and test all raw materials, including corn, at the scale with a validated method from FGIS. ♦ AFL monitoring strategy is in place based on the initial risk assessment done annually. ♦ Have dedicated paths for AFL‐contaminated grain based on end users and tolerance limits. If a given species' tolerance limit is exceeded, divert to another end user. AFL concentrates in dried distillers’ grains up to three times the original corn sample.	♦ Binder, 2007 ♦ Campbell et al., 1986 ♦ Marshall et al., 2020 ♦ Murthy et al., 2005 ♦ Scheid, 2012
Processing of corn at the grain processor or feed mill	♦ Frequent and regular cleaning of equipment based on manufacturer instructions and cGMPs. ♦ Monitoring program for manufacturing control procedures: cGMP, HACCP, ISO, etc. ♦ Have routine inspections, schedule samples and analysis, and turnaround time of stored grains. ♦ Physical methods: clean and sort corn using air or gravity separators, sieves, wet flotation, color sorting, dehulling, milling, or separating the outer seed coat.
	♦ Thermal methods: dry heating, superheated steam, extrusion cooking, and irradiation. ♦ Biological methods: bacteria, fungi, yeast, and other detoxification activities.	♦ Campbell et al., 1986 ♦ Coradi et al., 2016 ♦ Marshall et al., 2020 ♦ Scheid, 2012 ♦ Xu et al., 2022
End users (livestock)	♦ Use high‐quality and selected feed or food products. ♦ Test and monitor for AFL levels in feed ingredients. ♦ On‐farm management strategies: reduce exposure time, target feeds to species according to regulatory limits, and prevent by using feed additives (i.e., binders or bio‐transforming agents).	♦ Kaale et al., 2021 ♦ Munkvold et al., 2019 ♦ Neme & Mohammad, 2017 ♦ Park & Liang, 1993 ♦ Shabeer et al., 2022

Abbreviations: cGMPs, current good agricultural and manufacturing practices; FGIS, Federal Grain Inspection Service; HACCP, Hazard Analysisitical Control Point; ISO, International Organization for Standardization; PCQI, preventative controls qualified individual.

**FIGURE 2:**
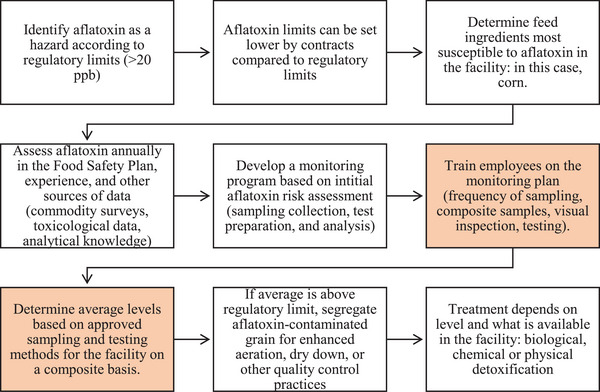
Food Safety Modernization Act (FSMA) tasks for aflatoxin (AFL) control in the feed and food supply chain. The highlighted orange boxes are areas for greatest improvement. Adapted from Fumagalli et al. (2021).

Based on the given strategies to manage AFL in FSMA‐regulated entities, Fumagalli et al. (2021) discussed that prevention or decontamination strategies for each feed chain phase, integrated into feed safety management systems, need to be established. Therefore, Figure 2 showcases a schematic with management strategies for AFL at FSMA‐regulated entities in Iowa based on SPFs within the system.

## 4 DISCUSSION

This study utilized historical Iowa AFL contamination data and an ETA to understand the probabilities of pivotal events with AFL in corn at FSMA‐regulated entities through feed safety systems. The goal was to provide an in‐depth understanding to guide proposed recommendations post‐harvest if AFL was present at the point of first receipt in Iowa corn above regulatory limits (>20 ppb). The probability of an IE with AFL‐contamination over 20 ppb identified by the data was 2.30% (Branstad‐Spates, Castano‐Duque, et al., 2023). It is important to note that AFL contamination changes annually, depending on many factors, including climate parameters, crop developmental markers, and weather conditions (Payne & Widstrom, 1992; Shotwell, 1977). Iowa's grain elevators, processors, and feed manufacturers are unique; therefore, there are multiple possible outcomes for AFL contamination in these facilities. Thus, this research was framed as a case study for an average of over 5 years in Iowa corn, and it would need to be updated annually with a food safety team in a food safety plan (FSP) for an FSMA‐regulated facility (Wester, 2017).

The goal of an ETA is to evaluate the probability of occurrence of each outcome (Ostrom & Wilhelmsen, 2019). In this case, the occurrence of interest was how AFL is received into an FSMA‐regulated facility and the potential outcomes of each pathway based on employee decision‐making and system characteristics. The ETA was able to evaluate non‐safety systems, including grain handling and feed manufacturing. Such systems are like safety systems in that they have a set of countermeasures intended to stop or reduce the effect of the consequences of the IE (Wallace et al., 2018). A safety system fails if countermeasures do not function or function appropriately (Ferdous et al., 2009). In contrast, if the countermeasures are a functional part of the system design and operate as designed, the safety system is considered successful. ETA explores each step of system component failure and illustrates how system countermeasures work successfully to prevent the IE from causing damage or injury (Ferdous et al., 2011). As one example, an ETA can assess how well countermeasures work to address or prevent AFL outbreaks in animal food or feed that can lead to adverse health events (Santos et al., 2014). This type of analysis is commonly used in emergency response systems, assessing new or improved operating procedures and considering management decision options. However, it has not been applied to the case of AFL in corn for the intent of feed products (Ahmadi & Soderholm, 2008; Lee et al., 2018; Rosqvist et al., 2013).

This case study allows stakeholders and researchers to easily see where the most significant probability of system failure exists in feed management systems concerning AFL in corn. In this study, the ETA shows the most significant likelihood of a failure occurring at sampling. Grain elevator, feed manufacturers, or processor PCQIs (or employees) are gatekeepers for AFL hazard management post‐harvest in the United States at FSMA‐regulated facilities. These individuals are the primary decision makers for testing inbound grain from field, being the point of first receipt of understanding AFL contamination before the rest of the supply chain (Park et al., 1999; Piñeiro, 2008). Some individuals may be temporary or seasonally hired for harvest and are most likely trained for efficiency and high throughput versus AFL and hazard management (Liu et al., 2016; Piñeiro, 2008). The extent of knowledge of AFL content in every lot of corn passing through the facility depends on its position in the supply chain and end use. Therefore, the ETA allows stakeholders and researchers to target better educational intervention, communication, and training toward system components with the most significant probability of failure in feed management systems. ETA cannot serve as the only component of system safety; however, it plays a vital role in identifying successes and failures for further analysis (Mosher & Keren, 2011). Using ETA and the historical AFL contamination data from Iowa in this scenario helped determine AFL risk and what decision scenarios should be made, determined by success and failure rates.

A potential application of the ETA is the ability to determine risk based on the overall consequences within the feed safety systems. A risk rating, which indicates the degree of risk for end users, can be estimated using the frequency and criticality of the event (Hong et al., 2009). A risk assessment matrix can be employed in an FSP through different techniques, such as failure mode and effects analysis, to identify and address the potential failures and effects on the system or process before an adverse event occurs (Gupta et al., 2022). Dependent on the failures, a preventative control (PC) may be necessary for AFL management if the risk assessment is high enough, as mandated by FSMA (Kheradia & Warner, 2013). Applying a PC would reduce AFL to a manageable level and reduce SPFs within the feed safety systems (Grover et al., 2016). This is an example of one strategy to better understand the risk of AFL levels, with the ability to control facility‐specific testing, handling procedures, and employee training post‐harvest.

Even though this scenario was able to provide a completed ETA with sound probabilities from previous literature, expert knowledge, and data, several limitations exist with this methodology. The objectives were narrowly defined in this scenario to AFL in Iowa corn post‐harvest; however, the outcomes can be challenging to illustrate graphically in an ETA (Mosher & Keren, 2011). There are multiple possible outcomes for AFL contamination in FSMA facilities dependent on the individuals working, training, operational equipment available, and what product is being produced for specific end users. It is impossible to trace using one ETA (Mosher & Keren, 2011). The data used for PCQI/employee's actions were based on the specific individuals who completed a survey in 2019, and it cannot be generalized to entire population groups (Bowers & Mosher, 2022). The data used do not account for interactions between systematic countermeasures and human responses, as it was used from a hypothetical standpoint (Mosher & Keren, 2011).

Secondly, the calculations of system success of mitigating AFL at 50.14% and the probability of failure at 49.86% are a case study scenario. Despite the high failure rate of mitigating AFL, these calculations potentially overestimate the actual value of what is happening in Iowa. Even though Iowa is not prone to large amounts of AFL contamination in corn, these events do exist with outbreak years due to drought and extreme weather conditions (Branstad‐Spates, Bowers, et al., 2023). Given climate change and northern latitudes being more prone to AFL in the future, risk management strategies such as an ETA need to be established at FSMA‐regulated facilities (Yu et al., 2022). Therefore, this study highlights that more testing and standardized mycotoxin management procedures are warranted to understand the accurate picture of what is happening at FSMA‐regulated facilities for AFL risk.

The end use of commodity corn is generally unknown, and action levels for AFL vary based on species, feed production, and operational use (Bryden, 2012). It is not feasible to sample and test every truckload of inbound corn for AFL during harvest due to the variability of sampling, time constraints, and economic impacts of testing (Whitaker, 2006; USDA‐ERS, 2023). If AFL passes into feed manufacturing facilities, it can evade mechanical, chemical, and thermal processing, making it nearly impossible to eradicate (Kępińska‐Pacelik & Biel, 2021). Thus, analyzing and predicting PCQI and employee decision‐making and preventative measures within feed safety systems post‐harvest operations is one systematic approach to quantifying AFL adverse events in Iowa, thus improving food and feed safety in the remaining portion of the supply chain (Krska et al., 2005). The ETA presented in this study quantifies risks that emerge because of specific actions taken by employees and their implementation of preventive measures. The specific pathways analyzed in this study do not represent all the potential scenarios or outcomes in the commodity corn supply chain. A key limitation of ETA is its ability to address a single event, and that limitation is true in this case.

## 5 CONCLUSION

This case study presents a novel approach combining historical Iowa AFL contamination data and an ETA to determine the success and failure of outbreaks in feed safety systems in FSMA‐regulated entities post‐harvest. Additionally, recommendations were created to assist employees and/or PCQIs with AFL management and monitoring at the point of the first receipt with corn. The probability of system success was 50.14%, and the probability of failure was 49.86%. The future holds increasing concerns about AFL, and customer demands will increase in proportion to knowledge. Proactive and preventative risk management strategies are the best way to combat AFL in feed systems. This proposed management methodology can be applied to other regions in the United States and with other handling and receipt scenarios to determine the risk of AFL and how to effectively manage contaminated corn and corn byproducts.
